# Tryptophan-Derived Uremic Toxins and Thrombosis in Chronic Kidney Disease

**DOI:** 10.3390/toxins10100412

**Published:** 2018-10-12

**Authors:** Tawfik Addi, Laetitia Dou, Stéphane Burtey

**Affiliations:** 1 Aix Marseille University, INSERM, INRA, C2VN, 13005 Marseille, France; tawfik.addi@gmail.com (T.A.); laetitia.dou@univ-amu.fr (L.D.); 2 LPNSA, Département de Biologie, Université d’Oran 1 Ahmed Benbella, 31000 Oran, Algérie; 3 Centre de Néphrologie et Transplantation Rénale, AP-HM, 13005 Marseille, France

**Keywords:** uremic toxins, thrombosis, chronic kidney disease, tryptophan

## Abstract

Patients with chronic kidney disease (CKD) display an elevated risk of thrombosis. Thrombosis occurs in cardiovascular events, such as venous thromboembolism, stroke, and acute coronary syndrome, and is a cause of hemodialysis vascular access dysfunction. CKD leads to the accumulation of uremic toxins, which exerts toxic effects on blood and the vessel wall. Some uremic toxins result from tryptophan metabolization in the gut through the indolic and the kynurenine pathways. An increasing number of studies are highlighting the link between such uremic toxins and thrombosis in CKD. In this review, we describe the thrombotic mechanisms induced by tryptophan-derived uremic toxins (TDUT). These mechanisms include an increase in plasma levels of procoagulant factors, induction of platelet hyperactivity, induction of endothelial dysfunction/ impairment of endothelial healing, decrease in nitric oxide (NO) bioavailability, and production of procoagulant microparticles. We focus on one important prothrombotic mechanism: The induction of tissue factor (TF), the initiator of the extrinsic pathway of the blood coagulation. This induction occurs via a new pathway, dependent on the transcription factor Aryl hydrocarbon receptor (AhR), the receptor of TDUT in cells. A better understanding of the prothrombotic mechanisms of uremic toxins could help to find novel therapeutic targets to prevent thrombosis in CKD.

## 1. Introduction

Chronic kidney disease (CKD) is defined as the occurrence of abnormalities of kidney structure or function, present for >3 months [1]. A decreased glomerular filtration rate (GFR) to less than 60 mL/min per 1.73 m^2^ is the commonest manifestation of CKD [2]. The decline of GFR is related to a substantial risk of mortality and cardiovascular disease (CVD) [3,4,5,6]. CVDs such as congestive heart failure, coronary artery disease, cerebrovascular disease, peripheral arterial diseases, atrial fibrillation, and sudden cardiac death represent cardiovascular pathologies that occur in patients with CKD [5,6].

Thrombosis is in the heart of the three major cardiovascular events: Ischemic stroke, ischemic heart disease, and venous thromboembolism [7]. CKD patients display an increased thrombotic risk, paradoxically associated with a bleeding tendency [8,9]. In CKD, thrombotic events occur in the cerebral, coronary, and retinal arteries, in the deep venous system, heart, and at sites of vascular access in hemodialysis patients [10]. Thrombus formation in arteries is often associated with atherosclerosis [8]. The risk of pulmonary embolism and deep vein thrombosis is significantly increased in CKD patients [11]. Moderate decrease in kidney function (30 < eGFR < 60 mL/min per 1.73 m^2^) and severe decrease in kidney function (eGFR < 30 mL/min per 1.73 m^2^) are respectively associated with a 2.5-fold and a 5.5-fold increase in venous thrombosis risk [12]. CKD patients display a higher mortality rate than patients with normal kidney function due to pulmonary embolism [8]. In CKD patients, the atrial fibrillation risk is higher and associated with an increased neurovascular risk [13]. In hemodialysis patients, thrombosis can complicate stenosis, leading to access failure [14].

## 2. Uremic Toxins from Tryptophan Metabolism

CKD leads to the accumulation of uremic toxins [15]. This accumulation exerts a deleterious effect on multiple organs/systems [15]. In 2003, the European Uremic Toxin Work Group (EUTOX) classified 90 uremic compounds. The number of compounds has been extended since [15]. The classification of uremic toxins includes three groups: Small water-soluble molecules (≤500 Da); middle molecules (>500 Da); and protein-bound molecules [15].

Some uremic toxins result from tryptophan metabolism through the indolic and the kynurenine pathways [16]. Dietary tryptophan is metabolized by the gut microbiota via three major pathways, leading to serotonin, kynurenines, and indole derivatives [16]. The kynurenine pathway that metabolizes 95% of tryptophan is mediated by enzymes tryptophan 2,3-dioxygenase (TDO) and indoleamine 2,3-dioxygenase (IDO). The activity of IDO leads to kynurenine from tryptophan and is reflected by the kynurenine/tryptophan ratio (KTR). Then, various metabolites can derive from kynurenine (KYN): 3-hydroxyanthranilic acid (3-HAA), 3-hydroxykynurenine (3-HKYN), kynurenic acid (KYNA), anthranilic acid (AA), and quinolinic acid (QA) [16]. The concentration of all kynurenine metabolites is increased in CKD patients, while tryptophan decreases. The indolic pathway leads to the indolic uremic toxins indoxyl sulfate (IS), indoxyl-β-d-glucuronide, and indole-3-acetic acid (IAA) [16]. In this pathway, tryptophan is converted to indole by the gut microbiota and absorbed into the blood circulation, then oxidized and sulfated in the liver by the microsomal p450 cytochrome CYP2E1 enzyme and sulfatase (SULT1A1) to form IS [17]. IAA is directly produced in the gut from tryptophan metabolism, or endogenously in tissue via tryptamine [16]. Because they are protein-bound, IS and IAA are badly cleared by dialysis [18].

## 3. Tryptophan-Derived Uremic Toxins and Thrombotic Events

An increasing number of studies are highlighting the link between tryptophan metabolites and thrombotic events in CKD. Studies support a role of tryptophan-derived uremic toxins (TDUT) and vascular access thrombosis in hemodialysis patients. Using multivariate Cox regression analysis in 306 patients undergoing endovascular interventions for dialysis access dysfunction, Wu et al. reported the amount of free IS predicts vascular access thrombosis, suggesting serum IS is a new risk factor for post-angioplasty thrombosis [19]. In 473 patients with advanced CKD, Chitalia et al. also demonstrated that patients with subsequent arteriovenous fistula (AVF) thrombosis had higher levels of IS, KYN, and KYNA than those without thrombosis [20]. Studies also showed the association of TDUT with cardiovascular events in CKD patients. KYN, 3-HKYN, KYNA, and KTR plasma concentrations are associated with increased risk of myocardial infarction [21,22,23]. In a cohort of CKD patients, Barreto et al. have shown IS predicts cardiovascular mortality [24]. In 120 patients with CKD, we demonstrated serum IAA predicts cardiovascular events and mortality, even after adjustment for CKD stage [25].

TDUT have an impact on thrombus formation. In Wistar rats, Karbowska et al. demonstrated that IS increases the weight of thrombus after vascular injury [26]. In mice exposed to high doses of IS, greater thrombus formation was observed after laser-induced endothelial injury, compared to non-IS exposed mice [26]. Another study showed that KYN enhances thrombosis after vascular injury in mice [20]. Chitalia’s group demonstrated that treatment of vascular smooth muscle cells (vSMC) by IS or IAA increases their thrombogenicity [27]. An important clot formation was observed in primary human vSMC pretreated with IS or IAA when exposed to coronary-like blood flow in an ex-vivo flow loop model [28].

## 4. Thrombotic Mechanisms Induced by Tryptophan-Derived Uremic Toxins

After an endothelial lesion or a blood vessel injury, subendothelial components are exposed to the blood and activate the process of hemostasis to initiate a blood clot formation [29]. The platelet plug formation involves platelet activation, adhesion to exposed subendothelial matrix components, and platelet aggregation [29]. Several platelet receptors and adhesive proteins, like the von Willebrand factor (vWF), fibrinogen, or collagen, as well as platelet-derived agonists, are involved in the interactions that normally occur only in the case of a vessel injury [29]. At the site of injury, the tissue factor (TF) from the vessel wall initiates the extrinsic pathway of the coagulation cascade. The complex TF/factor VIIa leads to the generation of factor Xa, which proteolytically cleaves prothrombin to form thrombin. Thrombin then mediates fibrin generation and platelet activation [29]. The coagulation cascade is also down-regulated by different factors, such as serine protease inhibitors antithrombin and tissue factor pathway inhibitor (TFPI) [29]. The deposition of insoluble fibrin and its incorporation into and around the platelet plug strengthen and stabilize the blood clot [29]. The fibrinolysis pathway, which breaks down the fibrin clot, also plays a substantial role in hemostasis [29]. In this pathway, tissue plasminogen activator (t-PA) released by the injured endothelium, and urokinase (uPA) in the presence of its receptor (uPAR) convert plasminogen to plasmin, which cleaves and destroys fibrin. Within the clot, t-PA activates plasminogen, which decomposes the fibrin mesh. t-PA and urokinase are inhibited by plasminogen activator inhibitor-1 and -2 (PAI-1 and PAI-2) [29].

A controlled balance between procoagulant and anticoagulant systems is essential, and a dysregulation can lead to pathological bleeding, or pathological thrombus formation, that is, thrombosis [29]. CKD, as well as TDUT, induce a profound deregulation of the mechanisms of hemostasis that affects all the players involved. 

### 4.1. Deregulation of Plasma Coagulation Factors

Several abnormalities in plasma factors of hemostasis are reported in CKD patients. These abnormalities are in favor of a hypercoagulable state, with enhanced levels of vWF, fibrinogen, thrombin, factor VII, TF, and PAI-1 [10,30], as well as reduced antithrombin activity [30]. In patients with CKD, we found that the concentration of circulating TF positively correlates with plasma levels of IS and IAA [31]. IS level also correlates with TF procoagulant activity [32] and with vWF in CKD patients [33]. Kynurenine is positively associated with vWF [34] and with prothrombin fragments F(1+2), reflecting hypercoagulability in CKD patients [35]. AA is also significantly associated with TF and prothrombin fragments F(1+2) [35], and negatively correlated with the fibrinolytic system uPA/uPAR in severe-to-end-stage CKD [36]. QA is positively associated with vWF [34], TF, and prothrombin fragments F(1+2) [35]. 3-hydroxykynurenine is positively associated with vWF [34] and with the TF/TFPI system [37] in CKD patients.

### 4.2. Platelet Hyperactivity

CKD is associated with both platelet dysfunction and activation [38,39]. Platelet activity is increased in CKD mice that display increased IS levels [40]. Ex vivo and in vitro, IS increases platelet activity, including an enhanced response to collagen and thrombin, increase in platelet microparticle production, and increase in platelet–monocyte aggregates [40]. In addition, IS-mediated platelet activation takes part in thrombus formation ex vivo and in vivo [40]. Reactive oxygen species (ROS)-induced p38MAPK signaling in platelets plays a crucial role in IS-induced platelet activation [40]. The inhibition of the ROS/p38 activation pathway by the ROS scavenger *N*-acetylcysteine (NAC) suppresses IS-induced platelet hyperactivation and platelet–monocyte aggregates, and strikingly attenuates IS-induced thrombus formation [40]. 

### 4.3. Endothelial Dysfunction

The vascular endothelium is essential for maintaining hemostasis and preventing thrombosis [41]. Healthy endothelial cells maintain an anticoagulant surface by expressing molecules that prevent platelet aggregation and fibrin formation [41]. When dysfunctional or activated, the endothelium can become prothrombotic and proinflammatory [41].

In CKD patients, endothelium dysfunction begins in the early stages of CKD [42] and takes a substantial part in thrombotic events [43]. In hemodialysis patients, endothelial injury is associated with an increased risk of AVF thrombosis [44,45,46]. The modifications of hemodynamic stress in vascular access and the accumulation of uremic toxins play a major role in endothelium dysfunction [45]. Tryptophan-derived indolic toxins induce a profound endothelial dysfunction, characterized by a procoagulant, pro-oxidant and proinflammatory phenotype [16,25]. In case of injury, these toxins could impair endothelial healing and vessel repair by inhibiting endothelial proliferation and migration, inducing endothelial senescence [16], and promoting progenitor cell apoptosis [44]. Another aspect of endothelial function is the generation of pro- and anticoagulant prostaglandins, which play an important role in hemostasis. Notably, endothelial cells produce Prostaglandin E2 (PGE2), which is generated by cyclooxygenases (COX) from arachidonic acid. PGE2 elicits platelet aggregation via its binding on platelet EP3 receptors and promotes thrombosis [47,48]. We demonstrated that IAA induces COX-2 expression in cultured endothelial cells via an aryl hydrocarbon receptor (AhR)/p38MAPK/Nuclear Factor kappa B (NF-κB) pathway [25]. Importantly, this increase in COX-2 enhances endothelial PGE2 synthesis [25], suggesting that IAA could promote platelet aggregation via endothelial synthesis of COX-2/PGE-2.

### 4.4. Decrease in NO Bioavailability

Nitric oxide (NO) released from endothelium and platelets prevents platelet adhesion to the vessel wall and inhibits platelet recruitment, aggregation, and activity [49]. NO supplies a negative feedback mechanism for thrombus propagation, and bioactive NO deficiency is associated with platelet dysfunction and arterial thrombosis [49]. CKD patients display NO deficiency, mainly to high concentration of the endogenous NO Synthase (NOS) inhibitor asymmetric dimethylarginine (ADMA), and defects in NOS activity [50]. Importantly, the increase in oxidative stress in CKD patients further reduces NO bioavailability [50]. Reactive oxygen species (ROS) have many effects on NO bioavailability: ROS increase ADMA levels, the ROS superoxide can react with NO to produce peroxynitrite that depletes the bioactivity of NO, and ROS oxidize tetrahydrobiopterin (BH4), which is crucial for eNOS activity [50]. Tryptophan-derived indolic toxins affect NO production: IS inhibits NO production in endothelial cells [51], but increases it in mononuclear cells [52]. In parallel, these toxins increase ROS production in blood mononuclear cells [52] and in vascular endothelial cells [25,53] by increasing endothelial NAD(P)H oxidase activity and decreasing the cellular amount of the antioxidant glutathione [53]. Thus, by favoring a pro-oxidant environment, indolic toxins could decrease NO bioavailability and thus contribute to increased platelet reactivity.

### 4.5. Production of Procoagulant Microparticles

CKD patients display high plasma levels of microparticles from platelet and endothelium [54,55]. Microparticles are membrane fragments that result from the remodeling of plasma membrane in response to activation or apoptosis [55]. Microparticles expose on their surfaces both phosphatidylserine, which facilitates the transformation of prothrombin to thrombin; and TF, which leads to thrombin generation, thus conferring a procoagulant potential [56,57]. Microparticles exhibiting TF are able to form a thrombus in vivo [58]. In vitro, we have shown that IS significantly enhances the release of microparticles by the endothelium [54]. Importantly, microparticles from endothelial cells incubated with indolic toxins exhibit a higher procoagulant activity [31]. Gao et al. also reported that IS and IAA enhance the procoagulant activity of red blood cells through releasing microparticles and exposing phosphatidyserine [59].

### 4.6. Overexpression of Cell Tissue Factor

TF induction in vascular cells by TDUT could be an important prothrombotic mechanism. TF is constitutively expressed in vascular wall cells, like fibroblasts and vSMC, to reduce bleeding after a vascular lesion [41]. TF is usually not expressed in endothelium, but activated endothelium and leukocytes could express active TF after a vascular lesion or inflammation [41]. Blood-borne TF comes mainly from leukocytes, platelets, and TF-bearing microparticles or circulates as a soluble protein [60]. TF exists as an encrypted form, with little or no procoagulant activity, which becomes activated (decrypted) upon vascular stimulation or injury [41].

In human aortic smooth muscle cells (SMC) that constitutively express TF, IS and KYN, as well as uremic serum, induce TF overexpression and increase its activity [20,27,61,62]. A prior treatment with an anti-TF neutralizing antibody reduces clot formation in vSMC exposed to coronary flow [27]. We reported that indolic toxins IS and IAA also induce TF expression in various types of cultured human endothelial cells that usually not express TF [31,63], as well as in leukocytes [31]. IS and IAA exacerbates the procoagulant activity of TF in endothelial cells and in endothelial microparticles [31]. In vivo, TF activity is greater in CKD patients with subsequent arteriovenous thrombosis than in those without thrombosis [20]. 

We demonstrated that TF induction by IS and IAA in endothelial cells occurred via a new pathway, dependent on the transcription factor AhR [31]. AhR is a receptor of some environmental contaminants, like 2,3,7,8-tetrachlorodibenzo-p-dioxin (TCDD) [64]. Indolic toxins IS, IAA, as well as kynurenines are potent endogenous agonists of AhR [16,65]. In resting cells, AhR forms a complex in the cytoplasm with HSP90, XAP2, and p23 [65]. Activation of the AhR genomic pathway by ligand binding causes AhR release from the heterodimeric complex and its interaction with its nuclear translocator ARNT (Aryl Hydrocarbon Nuclear Translocator) [65]. In the nucleus, the AhR/ARNT heterodimer recognizes specific XRE (Xenobiotic Response Elements) sequences on the promoters of AhR target genes, like genes of the cytochrome P450 family: CYP1A1, CYP1A2, or CYP1B1 [65]. Once activated by ligand, AhR can also cross-talk with multiple signaling pathways and elicit inflammation through a non-genomic pathway that does not require ARNT [66]. This non-genomic inflammatory pathway of AhR is involved in the induction of inflammatory genes, such as IL-1, IL-8, MCP-1, and COX-2 [25,67].

In endothelial cells, AhR inhibitors and AhR si RNA completely inhibit TF induction by indolic toxins [31,63]. We further showed that IAA induces endothelial TF expression via a non-genomic inflammatory pathway of AhR that induces p38MAPK and NF-kB activation [63]. In vSMC, IS extends TF half-life by reducing TF ubiquitination [27]. Shivanna et al. demonstrated that AhR activated by IS directly interacts with and stabilizes functional TF [32]. AHR regulates TF through STUB1, an ubiquitin ligase, which interacts with TF and degrades it through ubiquitination [68]. Consequently, IS thrombogenicity is significantly abrogated by the AHR antagonist in a flow loop ex vivo model [32]. KYN also increases TF expression via AhR in primary human aortic SMC [20] and regulates thrombosis after a carotid lesion in mice in an AhR-dependent way [20]. We demonstrated that patients and mice with CKD have high amounts of AhR agonists associated with cellular activation of AhR [69]. In 116 CKD patients, we further observed that cardiovascular events are more frequent in patients with high levels of AhR agonists [69]. In CKD patients, Shivanna et al. demonstrated IS levels correlate with AHR-inducing activity of serum and with TF activity [32]. CKD patients with arteriovenous thrombosis have a significant greater activity of AHR and TF than those without thrombosis [20]. AhR plays an important role in platelet production [70,71] and function [72]. The activation of the AhR/p38 MAPK pathway was observed in platelets in response to AhR agonists and resulted in enhanced platelet aggregation [73]. Therefore, AHR activation by TDUT could affect primary hemostasis, in addition to activation of coagulation, and could be a new prothrombotic mechanism in CKD patients. Clinical studies are now necessary to demonstrate that targeting AhR activation could be a new therapeutic option for preventing thrombosis in CKD patients.

## 5. Modulation of Microbiota and Diet to Reduce TDUT Production

Considering that TDUT arise from the metabolization of dietary tryptophan by the gut microbiota [16] and that gut dysbiosis begins in the initial stages of CKD [74], modifications of diet and/or use of prebiotics, probiotics, and symbiotics is a tempting approach. Results on probiotic therapies in CKD are conflicting. In a recent study, probiotic supplementation failed to reduce plasma levels of IS, IAA, and inflammatory markers in HD patients [75], whereas it decreased serum levels of IS in another study [76]. In CKD patients, symbiotic therapy did not significantly reduce serum IS [77], but probiotics may be interesting to reduce inflammation [74]. Results from diet modifications seem more promising. A low protein-diet reduces plasma levels of IS and urine levels of IS, indoxyl glucuronide, KA, and QA in healthy subjects [78]. In hemodialyzed patients, an increase in dietary fibers reduces the plasma levels of IS [79]. Finally, a diet with a higher proportion of protein from plant sources is associated with lower levels of IS [80] and with lower mortality rates in patients with CKD [81].

## 6. Conclusions

**Figure 1:**
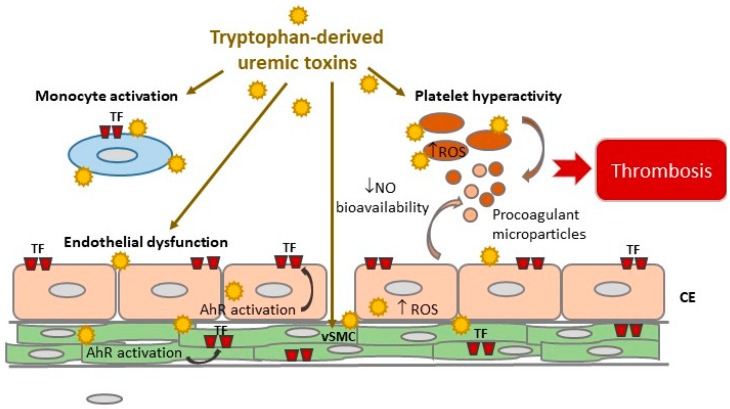
Mechanisms of tryptophan-derived uremic toxins promoting thrombosis. Tryptophan-derived uremic toxins promote thrombosis by inducing platelet hyperactivity, endothelial dysfunction, production of endothelial and platelet microparticles, and TF expression on monocytes, vSMC, and EC. TF induction is mediated by AhR activation. A decrease in NO bioavailability and an induction of ROS in platelets and endothelial cells participate in the thrombotic effects of tryptophan-derived uremic toxins. AhR: Aryl hydrocarbon Receptor; TF: Tissue Factor; EC: Endothelial cells, vSMC: Vascular smooth muscle cells; ROS: Reactive oxygen species; NO: Nitric Oxide.

TDUT accumulating in patients with CKD could induce thrombosis via several mechanisms: Activation of primary hemostasis and coagulation, endothelial dysfunction, decrease in NO bioavailability, and production of procoagulant microparticles (Figure 1). One important prothrombotic mechanism is the induction of tissue factor via AhR (Figure 1). This uremic toxins/AhR/TF axis may offer new therapeutic options to prevent thrombosis in CKD.
